# Parallel evolution and adaptation to environmental factors in a marine flatfish: Implications for fisheries and aquaculture management of the turbot (*Scophthalmus maximus*)

**DOI:** 10.1111/eva.12628

**Published:** 2018-04-06

**Authors:** Fernanda Dotti do Prado, Manuel Vera, Miguel Hermida, Carmen Bouza, Belén G. Pardo, Román Vilas, Andrés Blanco, Carlos Fernández, Francesco Maroso, Gregory E. Maes, Cemal Turan, Filip A. M. Volckaert, John B. Taggart, Adrian Carr, Rob Ogden, Einar Eg Nielsen, The Aquatrace Consortium, Paulino Martínez

**Affiliations:** ^1^ Department of Zoology, Genetics and Physical Anthropology University of Santiago de Compostela Lugo Spain; ^2^ CAPES Foundation Ministry of Education of Brazil Brasília Brazil; ^3^ Laboratory of Biodiversity and Evolutionary Genomics University of Leuven Leuven Belgium; ^4^ Center for Human Genetics UZ Leuven‐Genomics Core, KU Leuven Leuven Belgium; ^5^ Comparative Genomics Centre College of Science and Engineering James Cook University Townsville QLD Australia; ^6^ Faculty of Marine Science and Technology Iskenderun Technical University Iskenderun Turkey; ^7^ Institute of Aquaculture University of Stirling Stirling UK; ^8^ Fios Genomics Ltd Edinburgh UK; ^9^ Trace Wildlife Forensics Network Royal Zoological Society of Scotland Edinburgh UK; ^10^ National Institute of Aquatic Resources Technical University of Denmark Silkeborg Denmark

**Keywords:** adaptive variation, conservation genetics, population structure, RAD sequencing

## Abstract

Unraveling adaptive genetic variation represents, in addition to the estimate of population demographic parameters, a cornerstone for the management of aquatic natural living resources, which, in turn, represent the raw material for breeding programs. The turbot (*Scophthalmus maximus*) is a marine flatfish of high commercial value living on the European continental shelf. While wild populations are declining, aquaculture is flourishing in southern Europe. We evaluated the genetic structure of turbot throughout its natural distribution range (672 individuals; 20 populations) by analyzing allele frequency data from 755 single nucleotide polymorphism discovered and genotyped by double‐digest RAD sequencing. The species was structured into four main regions: Baltic Sea, Atlantic Ocean, Adriatic Sea, and Black Sea, with subtle differentiation apparent at the distribution margins of the Atlantic region. Genetic diversity and effective population size estimates were highest in the Atlantic populations, the area of greatest occurrence, while turbot from other regions showed lower levels, reflecting geographical isolation and reduced abundance. Divergent selection was detected within and between the Atlantic Ocean and Baltic Sea regions, and also when comparing these two regions with the Black Sea. Evidence of parallel evolution was detected between the two low salinity regions, the Baltic and Black seas. Correlation between genetic and environmental variation indicated that temperature and salinity were probably the main environmental drivers of selection. Mining around the four genomic regions consistently inferred to be under selection identified candidate genes related to osmoregulation, growth, and resistance to diseases. The new insights are useful for the management of turbot fisheries and aquaculture by providing the baseline for evaluating the consequences of turbot releases from restocking and farming.

## 1 INTRODUCTION

The detection of genetic structure in marine species represents a challenge due to generally high effective population sizes and high gene flow facilitated by the absence of physical barriers, which lead to genomic homogenization across populations (Danancher & Garcia‐Vazquez, 2011; Vandamme et al., 2014; Vilas et al., 2015). However, various factors can bring about genetic differentiation, such as habitat shifts (ecotones) and oceanic currents (Blanco‐Gonzalez, Knutsen, & Jorde, 2016; Galarza et al., 2009; Nielsen, Nielsen, Meldrup, & Hansen, 2004; Vera et al., 2016a), and natural selection in response to environmental variation (Milano et al., 2014; Vandamme et al., 2014; Vilas, Bouza, Vera, Millán, & Martínez, 2010; Vilas et al., 2015). Distinguishing between neutral and adaptive genetic variation has become a central issue in evolutionary biology, allowing for understanding of population structure in both historical/demographic and adaptive terms (Bernatchez, 2016; Nielsen, Hemmer‐Hansen, Larsen, & Bekkevold, 2009), thereby providing essential information for the conservation and management of wild populations. Genetic diversity in the wild represents, in turn, the raw material for the foundation of aquaculture broodstock and consequently, a reference to identify selection signatures for targeted traits in farmed populations through genome scanning (Liu et al., 2017).

The turbot, *Scophthalmus maximus* L., is a marine flatfish of the family Scophthalmidae, Order Pleuronectiformes, which lives in the Northeast Atlantic Ocean (from Morocco to the Arctic Circle) and in the Mediterranean Sea as well as in the Black Sea (Froese & Pauly, 2016). It has a demersal lifestyle and inhabits sandy coastal habitats (Bouza et al., 2014). Postlarval stages are relatively sedentary, with generally short migration distances (<10 km) being reported for both juvenile and adults of the species (Florin & Höglund, 2007; Nielsen et al., 2004). In contrast, the high dispersal potential of pelagic larvae (until ~30 days posthatching) mediated by oceanic currents coupled with the high fecundity provides potential for gene flow on larger spatial scales (Johannesson & André, 2006). Turbot is currently classified as a vulnerable species according to the IUCN European Red List assessment (Nieto et al., 2015). In some Atlantic regions, turbot fisheries are close to depletion and its main fisheries are located in the North Sea and in the Baltic Sea (ICES 2017a, 2017b), where turbot is exploited as a by‐catch species. An analysis of historical survey data in the North Sea suggests that turbot biomass has importantly declined, which might be associated with important biological changes in growth rate and reproduction (Bouza et al., 2014). In the Black Sea, the turbot is one of the most valuable commercial species and it is subjected to intensive fishing which has led to be characterized as exploited unsustainably and at risk of collapse (Nikolov et al., 2015). This scenario has led to restocking depleted areas with hatchery turbot with unknown outcomes, as its monitoring has not been accomplished (Bouza et al., 2014). On the other hand, as turbot breeding programs are at its beginning (Martínez et al., 2016), it is still feasible to introduce genetic variation from natural resources, especially in new geographical areas with particular environmental conditions. Although most turbot farms are located in the Atlantic area of Spain, France, and Portugal, its high commercial value is promoting new facilities in the Adriatic Sea (Croatia) and in the Black Sea (Turkey; FEAP, 2013).

Turbot experience a diverse physical and biological environment across its range. The Atlantic Ocean has a subtle salinity gradient running roughly from north to south, while sharp differences are found between the Northern Atlantic Ocean (≈35 PSU—practical salinity units) and the Baltic Sea (up to ≈2 PSU in the northern area; Environmental Marine Information System (EMIS) database; http://mcc.jrc.ec.europa.eu/emis/). Within the Baltic Sea, excluding the transition area with the North Sea, salinity can also reach higher values in the south (≈13 PSU). In the Mediterranean Sea, the salinity is even higher than in the Atlantic Ocean (≈38 PSU), but drops abruptly in the transition to the Black Sea, whose salinity levels resemble the Baltic Sea (≈11 PSU). Contrasting patterns of surface temperature also occur across latitude and between seasons. A north–south cline exists in the Atlantic area (annual average from ≈7°C in Norway up to ≈16°C off the Spanish coast), which increases further in the transition to the Mediterranean Sea (≈21°C), especially during summer. A sharp winter versus summer variation is also found within the inner Baltic and Black seas (≈20°C difference). In the latter, turbot was formerly described as a subspecies (*Psetta maxima maoetica*; Tortonese, 1971), but currently it is considered a geographical variant of *S. maximus* based on morphological and genetic data (Atanassov, Ivanova, Panayotova, Tsekov, & Rusanov, 2011; Bailly & Chanet, 2010; Blanquer, Alayse, Berrada‐Rkhami, & Berrebi, 1992; Bouza et al., 2014; Suzuki et al., 2004).

The uneven distribution of turbot has been associated with phylogeographic events related to rapid adaptive radiation following the last glaciation and to heterogeneous environmental conditions across its distribution range (Bouza, Presa, Castro, Sánchez, & Martínez, 2002; Vandamme et al., 2014). Some life‐history traits, such as growth, survival, reproduction, and fecundity, have been shown to be influenced by temperature (Felix, Vinagre, & Cabral, 2011). Previous population genetic studies have shown low or no genetic population structure over large geographical areas, such as in the Northeast Atlantic Ocean. This has been attributed to the advection of larvae and, in some cases, also to the active migration of adults (Bouza, Sánchez, & Martínez, 1997; Bouza et al., 2002, 2014; Coughlan et al., 1998). Genetic divergence has been documented to be mainly associated with isolated areas in the Western and Eastern Mediterranean Sea (Atanassov et al., 2011). Low but significant differentiation between the Atlantic Ocean and Baltic Sea turbot has also been reported (Florin & Höglund, 2007; Nielsen et al., 2004; Vilas et al., 2010).

Despite the overall high genetic homogeneity recorded for turbot, strong site fidelity to the spawning sites and limited dispersal of adults during the reproduction period have been documented in the Baltic Sea (Florin & Franzén, 2010), suggesting that geographical segregation, even within continuous areas, might occur. Additionally, evidence suggestive of adaptation to temperature and salinity at a regional level has been reported (Vandamme et al., 2014; Vilas et al., 2015). Reproductive success and growth differences in turbot between the Atlantic Ocean and the Baltic Sea have been associated with salinity (Nissling, Johansson, & Jacobsson, 2006) and have been explained either by phenotypic plasticity (Florin & Höglund, 2007) or divergent selection (Vilas et al., 2010, 2015). Significant divergence at specific markers (SNPs or microsatellites) has been reported between turbot sampled from the Irish shelf and the North Sea, the English Channel and the Biscay Gulf, between southern and northern North Sea, and between Baltic and North Sea (Vandamme et al., 2014; Vilas et al., 2010, 2015). An earlier study identified different hemoglobin genotypes, which suggested that turbot populations in the Northern Atlantic Ocean might not be entirely homogeneous (Imsland, Scanu, & Nævdal, 2003). These data highlight the need for more detailed studies using larger genomic coverage to clearly elucidate both neutral and adaptive genetic differentiation. Moreover, despite turbot being well‐studied, its population structure has not been explored across its full distribution range; knowledge on wild populations is mostly limited to the Atlantic and Baltic areas and genomic features associated with environmental variables have only been recently investigated in a limited number of populations (Vilas et al., 2010, 2015) and markers (Vandamme et al., 2014). With the turbot genome recently sequenced (Figueras et al., 2016) and the technology available to rapidly discover and survey hundreds of SNP markers (Robledo, Palaiokostas, Bargelloni, Martínez, & Houston, 2017), it is now practical to consider conducting a genomewide scan of turbot populations, in order to better describe and understand its population genetic structure.

In this study, a panel of 755 SNPs evenly distributed over the turbot genome were genotyped using the double‐digest RAD sequencing (ddRAD‐seq) technology (Peterson, Weber, Kay, Fisher, & Hoekstra, 2012) and used to screen populations at a large geographical scale to (i) evaluate the level of population differentiation at small and large scales, (ii) test whether similar environmental conditions led to parallel evolution/adaptation in geographically distant/independent populations, and (iii) test the discrimination power of neutral versus outlier markers to define turbot populations for later applications in traceability studies. The information gathered is useful for a sustainable management of genetic resources in the wild and for guiding selection of genetic raw material for the growing turbot aquaculture.

## 2 MATERIALS AND METHODS

### 2.1 Sampling

**Figure 1:**
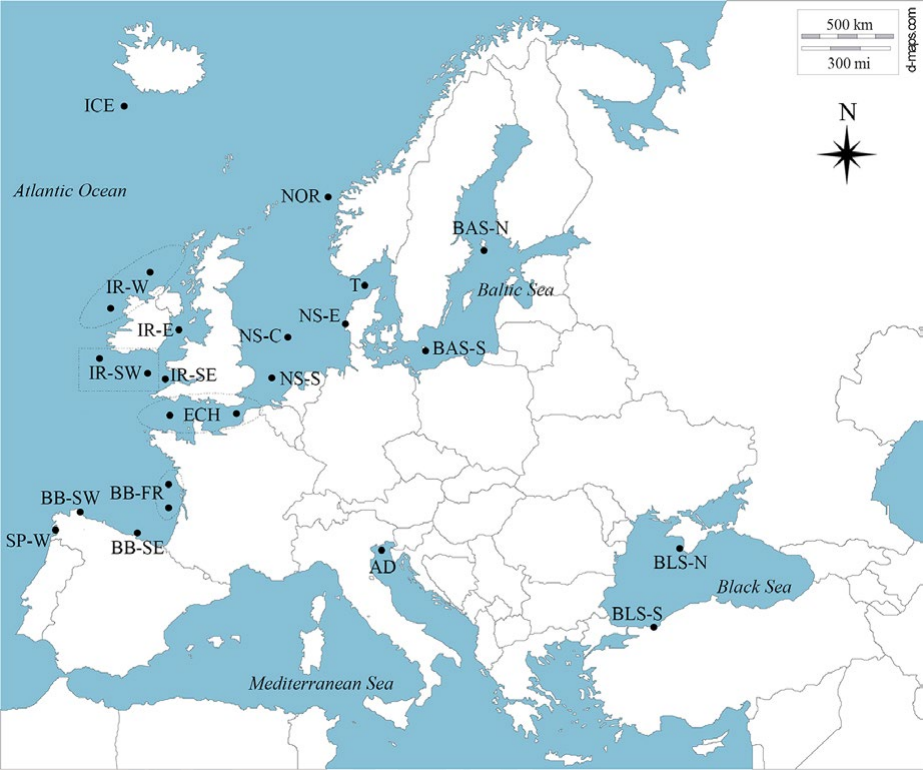
Geographical location of *Scophthalmus maximus* samples

A total of 697 individuals were collected at 20 sampling sites, mostly exceeding 20 individuals per sample, and often above 30 (Figure 1, Table 1). Sampling was carried out in the four main areas of the turbot distribution range: Baltic Sea (BAS), Atlantic Ocean (ATL), Mediterranean Sea (MED) and Black Sea (BLS). The geographical transitional area between the ATL and BAS (Skagerrak‐T) was also sampled. Most samples from ATL and BLS corresponded to archived samples collected during previous oceanographic campaigns and a few new ones from landings. Despite intensive sampling effort off the coasts of Spain, Italy and Greece (Murcia, SE Spain; Rosas, NE Spain; Ionian Sea and Adriatic Sea, Italy; and Aegean Sea, Greece), only one Mediterranean Sea sample was large enough for analysis (Adriatic Sea: AD; 37 individuals), symptomatic of the current scarcity of turbot in this area—possibly related to thermal constraints. Thus, most samples came from the Atlantic area (including the Baltic Sea) and only three were collected in the southeastern area, that is, the aforementioned sample from the Adriatic Sea and two sites from the Black Sea (Figure 1, Table 1). Samples were pooled according to ICES fisheries subdivisions (III, IV, V, VI, VII, VIII, IX) at some locations in the Atlantic area where fewer turbot were caught and previous studies had suggested genetic homogeneity (Vandamme et al., 2014), so, in summary, a total of 17 samples were investigated in the Atlantic area, two of them from the Baltic Sea and one from the transition North Sea‐Baltic Sea. Samples were taken throughout the different seasons of the year, about half during the breeding season (spring and summer; Bouza et al., 2014).

**Table 1:** Sampling characteristics of *Scophthalmus maximus*

Region	Sample location	Sample size	ICES	Pop ID	Sampling date	Spatial variables		Environmental variables				
						Lat	Long	SST	SBT	SSS	SBS	PP
Baltic Sea	Baltic Sea—North	33	IIId	BAS‐N	2003, 2013	60.2	19.7	8.3	5.6	6.1	5.5	3.3
Baltic Sea	Baltic Sea—South	45	IIId	BAS‐S	2000	55.0	14.9	7.2	9.0	8.2	13.5	2.6
Transition area	Skagerrak	38	IIIa	T	2001	57.4	9.2	8.1	13.8	33.7	21.6	2.1
Norway Sea	Norway Sea	19	IVa	NOR	2011	62.0	4.0	9.5	7.5	33.5	33.8	10.7
North Sea	North Sea—East	47	IVb	NS‐E	2001	56.4	6.1	12.5	8.4	34.4	34.8	5.4
North Sea	North Sea—Central	46	IVb	NS‐C	2010–2011	55.4	5.3	8.5	13.0	33.8	26.1	8.1
North Sea	North Sea—South	24	IVc	NS‐S	2009–2011, 2013	51.8	2.0	21.8	12.3	34.2	12.5	20.6
North Atlantic	Iceland	13	Va	ICE	2010–2011	63.2	−21.1	7.3	7.4	35.2	24.3	10.7
British Isles	Ireland—West	47	Vla. VIIb	IR‐W	2009–2011	59.3	−4.5	9.9	15.5	35.3	23.8	17.2
British Isles	Ireland—East	45	VIIa	IR‐E	2009–2010, 2013	53.5	−4.8	8.3	10.1	33.9	15.7	9.1
British Isles	Ireland—Southwest	22	VIIg. VIIj	IR‐SW	2009–2011	50.6	−6.0	15.0	14.7	34.1	26.0	9.8
British Isles	Ireland—Southeast	20	VIIf	IR‐SE	2009–2010	50.4	−5.8	11.0	15.5	34.1	18.2	10.2
English channel	English channel	18	VIId. VIIe	ECH	2009–2010	50.7	8.4	17.1	17.1	34.9	34.9	0.7
Biscay bay	Biscay bay—France	25	VIIIa. VIIIb	BB‐FR	2009–2010	46.2	−2.2	15.1	11.0	34.1	12.6	9.1
Biscay bay	Biscay bay—Southeast	48	VIIIc	BB‐SE	2013	43.4	−3.8	12.9	9.7	35.1	16.0	23.9
Biscay bay	Biscay bay—Southwest	41	VIIIc	BB‐SW	2002	43.7	−7.4	12.9	11.3	35.4	18.0	12.4
Spain	Spain coast—West	49	IXa	SP‐W	2002	42.6	−8.9	8.6	19.4	34.9	35.4	6.1
Mediterranean Sea	Adriatic Sea	37	37.2.1	AD	2013–2014	45.2	12.3	18.5	9.3	30.6	38.3	7.8
Black Sea	Black Sea North	25	37.4.2	BLS‐N	2009–2010	44.6	33.4	10.1	7.4	18.5	12.0	10.9
Black Sea	Black Sea South	30	37.4.2	BLS‐S	2013	41.1	31.1	20.5	6.6	18.3	11.0	17.1

ICES, region according to the International Council for the Exploration of the Seas; Lat, latitude; Long, longitude; SST, sea surface temperature; SBT, sea bottom temperature (°C); SSS, sea surface salinity (PSU); SBS, sea bottom salinity (PSU); PP, primary production (mg C m^−2^ day^−1^).

### 2.2 SNP calling and genotyping

A ddRAD genotyping‐by‐sequencing (GBS) approach was carried out following the procedure first described by Peterson et al. (2012) with slight modifications, detailed in full elsewhere (Brown et al., 2016). Five ddRAD libraries were made, each comprising a pool of 144 individuals, tagged using combinatorial and inline barcodes. A proportion of individuals from each sample was included in every library to control for technical library‐related scoring biases. Size selection in agarose gels aimed to retrieve amplified PCR fragments ranging from 190 base pairs (bp) to 460 bp. Libraries were sequenced on the Illumina 2500 platform at BMR Genomics (Padova, Italy), using 100‐bp pair‐end chemistry. Demultiplexing and quality filtering were undertaken using custom scripts developed by Fios Genomics (Edinburgh, UK). Read‐pairs with the expected restriction sites and full barcodes at both ends were identified, allowing up to one error in each barcode. After barcode trimming, all sequences had a uniform length of 90 bases. Read‐pairs with one or more uncalled bases or 11 or more consecutive bases with average Phred scores below 20 were excluded. SNP calling and genotyping was performed with STACKS software v1.30 (Catchen, Hohenlohe, Bassham, Amores, & Cresko, 2013) considering both read‐pairs as independent tags: The *ustacks* module was used for setting the bounded model to merge tags into loci (‐m 4, ‐M 7, –alpha 0.01), and then, a comprehensive catalog of loci was created using the *cstacks* module (‐n 7) and the full dataset. A correction of SNP calls was made using population data with the *rxstacks* module, which improves SNP calls and filters unreliable and confounded loci. Finally, the *populations* module was used to retrieve a first dataset of SNPs in *genepop* format for doing the final filtering step. Genotyping accuracy was validated using sample replicates from within and among libraries.

Following the initial SNP calling, loci that failed the following filtering criteria were removed from the dataset: (i) genotyped in >80% individuals, (ii) contained a single SNP per RAD‐tag, (iii) had a unique match against the turbot genome (Figueras et al., 2016) using BLAST (e‐value <1e‐20; Altschul et al., 1997), (iv) had a minimum allele frequency (MAF) >0.002, and (v) conformed to Hardy–Weinberg equilibrium (HWE), that is, loci with consistent and significant (*p *>* *.05) *F*_IS_ values (Weir & Cockerham, 1984) across populations. Two subsets of the final marker set after filtering were identified to assess genetic structure: presumed neutral SNPs (neutral dataset) and outlier SNPs (selection dataset; see subsection 3.1).

### 2.3 Genetic diversity and structure

Mean number of alleles per locus (Na), expected (H_E_), and observed (H_O_) heterozygosities and percentage of polymorphic loci at 95% (P_95_: frequency of the most common ≤0.95) and 99% (P_99:_ ≤ .99) were calculated to assess genetic diversity. H_E_ was also calculated for a set of the most polymorphic, potentially more informative, loci, that is, where MAF ≥ 0.05. Departure from Hardy–Weinberg equilibrium (HWE) and significance of *F*_IS_ values were tested for each population. Analyses were performed using GENEPOP v4.0 (Rousset, 2008) and ARLEQUIN v3.5 (Excoffier & Lischer, 2010) software. Effective population size was estimated by NeESTIMATOR v2.01 (Do et al., 2014) using the linkage disequilibrium (LD) method and loci with a MAF >0.02.

Pairwise *F*_ST_ values between sampling sites and between geographical regions were estimated with ARLEQUIN v3.5 and tested for significance using 10,000 permutations. To further investigate population structure, a Bayesian individual clustering approach was applied using the program STRUCTURE v2.3.4 (Pritchard, Stephens, & Donnelly, 2000) based on the admixture ancestry model and correlated allele frequencies. A burn‐in of 10,000 iterations was used, followed by a MCMC procedure (Markov chain Monte Carlo) of 50,000 repeats, and tested *K* (number of genetic clusters or units) from 1 to 10. Five independent runs were used for each *K* estimate when the full marker dataset was used, while 10 runs per *K* were tested for the neutral and outliers marker subsets. The *locprior* model, which specifies the population of origin of each individual, was also used, as previously suggested for data showing weak structure (Hubisz, Falush, Stephens, & Pritchard, 2009; Pritchard et al., 2000; Vandamme et al., 2014).

Results from STRUCTURE were processed with the program STRUCTURE HARVESTER v0.3 (Earl & vonHoldt, 2012) to estimate the best‐fitted number of clusters *K* based on the Δ*K* method described by Evanno, Regnaut, and Goudet (2005). However, as hierarchical analysis assumed by STRUCTURE may overshadow other *K*‐values and affect the detection of substructure, HARVESTER was also run excluding *K *=* *1. Under this scenario and for all tests, several *K* assignments were explored to better resolve subtle structuring, as previously suggested (Pritchard et al., 2000; Vandamme et al., 2014). Additionally, because STRUCTURE inference may be affected by uneven sampling (Puechmaille, 2016), as in our case due to the extensive sampling of the Atlantic area compared to other areas such as Adriatic and Black Sea, more restricted analyses including only Atlantic samples were performed to detect subtle genetic structuring. The software CLUMPP v1.1.2 (Jakobsson & Rosenberg, 2007) was used to estimate the most likely cluster membership coefficient among the different runs tested.

Discriminant analyses of principal components (DAPC) were run in the ADEGENET package (Jombart & Ahmed, 2011; Jombart, Devillard, & Balloux, 2010) written for the R platform (R Development Core Team, 2014; http://www.r-project.org). Data were transformed using principal component analysis (PCA) and an appropriate number of principal components and discriminant functions retained.

A Mantel test was performed using GENEPOP v4.0 to test for isolation by distance (IBD). The correlation between *F*_ST_/1–*F*_ST_ and Ln geographical distance was used for the option ISOLDE and 10,000 permutations in this same program. Minimum sea distances (km) between sampling sites were obtained using Google Earth (Google Inc, USA).

For all statistical tests, significance level (*p *<* *.05) was corrected for multiple comparisons using the sequential Bonferroni method (Rice, 1989).

### 2.4 Detection of outlier loci and mining of the turbot genome

To identify candidate loci subjected to selection, three different algorithms and related software packages were applied as previously suggested (Narum & Hess, 2011; Shimada, Shikano, & Merilä, 2011; Souche et al., 2015). BAYESCAN v2.01 (Foll & Gaggiotti, 2008) was used following 20 pilot runs, 5,000 iterations, 5,000 burn‐ins steps, and a sample size of 5,000. A log10 Bayes factor (BF) >2 (*p *>* *.99) was used as an initial threshold but log10 BF >1.3 (*p *>* *.95) was also evaluated for comparisons with other methods. BAYESCAN outcomes were plotted using the R functions provided by the program. The FDIST *F*_ST_ method implemented in the LOSITAN software (Antao, Lopes, Lopes, Beja‐Pereira, & Luikart, 2008) was used to investigate loss of heterozygosity (expected after selective sweeps) regarding *F*_ST_. For this program, a total of 100,000 simulations, a population size of 50, and the infinite allele mutation model were used. Analyses were run using a confidence thresholds of 0.99, a false discovery rate (FDR) of 0.01, and the “neutral mean *F*_ST_” option. ARLEQUIN v3.5 was applied to investigate outlier loci among groups of samples under a hierarchical island model, testing 20,000 simulations, 50 groups, and 100 demes per group. SNP markers with *p *<* *.05 or <.01 were considered as outlier loci depending on the analysis.

Taking into account the low structuring of turbot across its distribution range (Bouza et al., 2014), the identification of outliers for divergent selection would be expected more feasible and confident than those for stabilizing selection, especially in the Atlantic Ocean and Baltic Sea, the most representative regions of the species. Accordingly, all loci detected by any of the aforementioned methods were considered the initial set of candidates for divergent selection. A more stringent approach was used to identify divergent outliers with the highest confidence. Among the programs applied, BAYESCAN follows the most conservative approach and identifies a smaller number of markers than LOSITAN and ARLEQUIN, which usually return a high proportion of false positives (Narum & Hess, 2011; Shimada et al., 2011). Accordingly, all outlier loci detected with BAYESCAN (*p* < .050) were retained, and additionally, only those that were identified both with LOSITAN and ARLEQUIN at the highest confidence level (CI < 99 and FDR = 0.010 for LOSITAN and *p* < .010 for ARLEQUIN). Outlier loci for balancing selection are more difficult to detect, and a high rate of false positives is returned with most programs (Narum & Hess, 2011); this fact could be even stressed considering turbot structure. Thus, only markers detected with at least two of the three approaches were considered significant in our study, additionally considering the environmental scenario of the area analyzed (see Results).

Outliers were investigated using all samples or subgroups of samples according to the observed regional structure (see Results section and previous reports Vandamme et al., 2014; Vilas et al., 2010, 2015): ATL (14 populations excluding the Baltic‐Atlantic transition area); ATL & BAS (17); ATL & BLS (16); BAS & BLS (4); and BAS (2). The AD sample was not included in this analysis due to its limited representativeness of the Mediterranean area.

Sequences (90 bases) including the most consistent set of outlier SNPs were mapped to the turbot genome to obtain their genomic position using local BLASTn (e‐value <1e‐20). These outliers were subsequently anchored to the turbot genetic map (linkage group (LG) and predicted position in cM) to investigate their putative relationship with previously reported outliers (Vilas et al., 2010, 2015) or QTLs (Martínez et al., 2016). Gene mining around identified outlier loci was accomplished using the turbot genome browser ( http://denovo.cnag.cat/genomes/turbot/; Figueras et al., 2016). Gene lists were compiled along an interval of ±250 kb flanking the outlier locus position, this considered appropriate due to the low population linkage disequilibrium observed in turbot (Saura et al., 2017). Suggestive candidate genes involved in immunity and growth, or putatively related to environmental variables, along with those in the vicinity of previously described QTLs were retrieved from gene lists. Functional enrichment of the gene lists against the turbot genome was undertaken with BLAST2GO (Conesa et al., 2005).

### 2.5 Seascape analyses

The environmental variables “sea surface temperature” and “sea bottom temperature” (SST and SBT, respectively, in °C), “sea surface salinity” and “sea bottom salinity” (SSS and SBS, respectively, in PSU), and “primary production” (PP in g C m^−2^ day^−1^) were retrieved from the Environmental Marine Information System (EMIS) database ( http://mcc.jrc.ec.europa.eu/emis/) and the Copernicus database ( http://marine.copernicus.eu/), corresponding with the average along the year of the monthly mean values for each sampling site with a 4‐km resolution for the period comprising 1997–2014.

Genetic differentiation explained by spatial variables (i.e., latitude and longitude) and the registered environmental variables (SST, SBT, SSS, SBS, and PP) was studied by a canonical redundancy analysis (RDA) using the R platform VEGAN software (Oksanen, 2015). For each sample, allele frequencies were estimated for all analyzed loci with the ADEGENET package on the R platform using the “makefreq” option applied on the ADEGENET “genpop” file. Only two loci showed missing values to estimate allele frequencies, and consequently, they were removed for analyses as VEGAN does not allow missing information in their input files. ANOVA tests were performed to check the significance of the variance associated with the different environmental variables using 1,000 random permutations with VEGAN. Variance inflation factor (VIF) was estimated to explore collinearity (correlation) among environmental variables in our dataset. VIF values <5 show no collinearity problems, values from 5 to 10 represent moderate problems, and values >10 indicate important collinearity problems (Marquardt, 1970). Different models were adjusted by automatic forward selection based on significant variable criteria. These analyses were performed using the PACKFOR package in R (Dray, Legendre, & Blanchet, 2009). Forward selection corrects for highly inflated type I errors and overestimated amounts of explained variation (Vandamme et al., 2014). Thus, the reduced panel of explanatory variables was used to recalculate the total proportion of genetic variation in the variance partitioning. The weight of the different loci on the significant environmental vectors was obtained using VEGAN. All these analyses were performed separately for the full and the neutral SNP datasets.

## 3 RESULTS

### 3.1 Sequencing

A total of 783,829,288 read‐pairs were retrieved from the sequencing platform, with ~1 million read‐pairs on average per sample. The distribution of reads per sample was rather unbalanced, ranging from 125,563 to 5,842,110, mostly attributed to the low DNA quality of some archived samples, along with the specific methodology employed; that is, samples were combined immediately following adapter ligation (Brown et al., 2016) and not amplified, size selected and re‐quantified individually before pooling into a library as described for the original protocol (Peterson et al., 2012). After barcode and restriction enzyme site identification, an average of 85% of read‐pairs passed the first quality‐filtering step, leaving 659,968,515 sequences to feed into the STACKs pipeline. We developed a custom bioinformatic pipeline supported by STACKS software and the genomic resources of the target species. STACKS parameters were chosen after careful optimization aimed at using the best combination according to the species and the specific goals of the study. With this tool, a catalog with 524,421 loci was built, from which the *populations* module extracted a first dataset of 5,564 polymorphic loci. A final set of 755 SNPs was obtained after the application of the following filtering steps: (i) 3,656 loci did not pass the cutoff of genotyping coverage (>80%), (ii) 899 loci showed MAF <0.002, (iii) 40 loci showed consistent deviations from HWE across populations (*p *<* *.05), suggesting null alleles (*F*_IS_ > 0) or mixing of reads from different paralogs (*F*_IS_ < 0), (iv) 71 loci corresponded to the same SNP due to overlapping RAD‐tags, (v) 136 loci co‐mapped in the same RAD‐tag with another SNP, and (vi) 7 SNPs did not match with the turbot genome. Genotyping accuracy was evaluated in this filtered sample using the sample replicates within and among libraries, and an average genotyping error rate of 0.5% was recorded. A total of 25 individuals with very low DNA quality and genotyped for <20% of the SNP panel after filtering were discarded. The final panel of 755 SNP loci was analyzed in 672 individuals to evaluate genetic diversity and structure, identification of outlier loci and landscape analysis.

### 3.2 Genetic diversity

On average genetic diversity values across all populations amounted for Na to 1.49 ± 0.50, H_E_ to 0.093 ± 0.144, H_E_ (P_95_) to 0.189 ± 0.154, and P_99_ to 49.0% ± 2.1% (Table 2). Excluding the smallest collected sample (ICE), samples from BAS, AD, and BLS were the least diverse (Na from 1.28 to 1.45; H_E_ from 0.072 to 0.088; P_99_ from 28% to 45%). Likewise, a lower effective population size (N_e_) was estimated for these samples, especially for BLS‐N (126) and AD (46; Table 2). Samples from the Atlantic area showed the highest diversity (Na from 1.47 to 1.60; H_E_ from 0.092 to 0.096; P_99_ from 47% to 60%), and most N_e_ values were higher than 1,000 (many of them ∞ suggesting values above 5,000–10,000 considering the limitations of the LD estimator). Within the ATL region, the lowest N_e_ values were for IR‐E (264), BB‐SE (166) and SP‐W (411). Significant deviations from HWE, resulting from heterozygote deficit, were only found within the Atlantic/Baltic Sea area at BAS‐N (*F*_IS_ = 0.162) and BB‐SW (*F*_IS_ = 0.158; *p* < .050), but especially at BAS‐S (*F*_IS_ = 0.201; *p* < .050 after Bonferroni correction).

**Table 2:** Genetic diversity of *Scophthalmus maximus* throughout its geographical distribution

Pop code	Sample size	Na	H_E_	H_E_ (P_95_)	P_99_ (%)	*F*_IS_	N_e_
BAS‐N	33	1.45	0.088	0.198	45	**0.162**	730
BAS‐S	45	1.45	0.081	0.182	45	**0.201**	Infinite
T	38	1.55	0.092	0.166	55	0.122	796
NOR	19	1.47	0.098	0.210	47	0.066	Infinite
NS‐E	47	1.59	0.097	0.165	59	0.095	Infinite
NS‐C	46	1.59	0.095	0.160	59	0.084	Infinite
NS‐S	24	1.49	0.092	0.189	49	0.099	1468
ICE	13	1.38	0.092	0.243	38	0.153	Infinite
IR‐W	47	1.57	0.094	0.163	57	0.126	624
IR‐E	45	1.58	0.094	0.163	58	0.129	264
IR‐SW	22	1.49	0.093	0.192	49	0.103	Infinite
IR‐SE	20	1.48	0.097	0.204	48	0.055	Infinite
ECH	18	1.46	0.096	0.208	46	0.109	Infinite
BB‐FR	25	1.50	0.095	0.189	50	0.059	1813
BB‐SE	48	1.59	0.093	0.157	59	0.099	1733
BB‐SW	41	1.55	0.092	0.168	55	**0.158**	166
SP‐W	49	1.60	0.096	0.161	60	0.063	411
AD	37	1.43	0.087	0.203	43	0.083	46
BLS‐N	25	1.28	0.073	0.265	28	0.138	126
BLS‐S	30	1.31	0.078	0.257	31	0.069	489
	672	1.49	0.093	0.189	49	0.109	

A, mean number of alleles per locus; H_E_, expected heterozygosity; P_95_, percent of polymorphic loci (minimum allele frequency (MAF) ≥ 0.05); Ho, observed heterozygosity; HE (P_95_), expected heterozygosity calculated using polymorphic loci at P_95_; *F*_IS_, inbreeding coefficient; Ne, effective population size considering a lowest allele frequency of 0.02; in bold, *F*_IS_ *p* < .05; in bold and underlined, significant *F*_IS_ values after sequential Bonferroni correction (*p* < .0001).

### 3.3 Genetic structure

**Figure 2:**
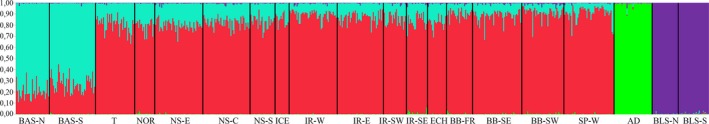
STRUCTURE results of all samples of *Scophthalmus maximus* for the most likely number of clusters (*K* = 4) computed using the complete 755 SNP panel

**Figure 3:**
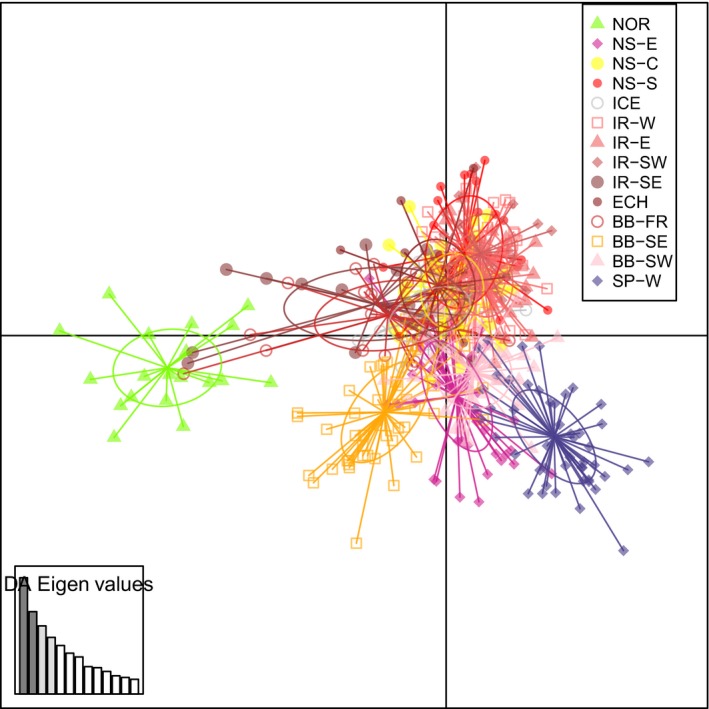
DAPC analysis of *Scophthalmus maximum* Atlantic samples computed using the complete 755 SNP panel

Moderate but highly significant genetic differentiation (*F*_ST_ = 0.090, *p *<* *.001) was detected across the whole turbot distribution using the 755 SNP panel, mostly attributable to divergence between the more isolated areas (Black Sea and the Adriatic Sea) and the Atlantic Ocean and the Baltic Sea (Table 3 and Table S1). The most likely number of genetic units (*K*) was four as revealed by STRUCTURE (i.e., ATL, BAS, AD, BLS; Figure 2 and Figure S1) and DAPC (Figure S2a), corresponding with the four main geographical sampled areas (Figure 1). The divergence was moderate–high between the southern (AD and BLS) and the Atlantic region (*F*_ST_ = 0.137 and 0.134, respectively; *p *<* *.001), and low, but significant, between the Baltic and the Atlantic region (*F*_ST_ = 0.005; *p *<* *.001; Table 3). All pairwise *F*_ST_ (Table S1), STRUCTURE (Figure 2 and Figure S3) and global DAPC (Figure S2a) analyses depicted samples from the ATL region as relatively homogeneous. However, subtle structure was detected in the Baltic Sea (BAS‐N vs. BAS‐S; Figure S2a) and in the Atlantic area (Figure 3), where samples from the northern (NOR) and southern (SP‐W and BB ‐SW) extremes plotted separately.

**Table 3:** Pairwise *F*_ST_ matrices for the four geographical areas of *Scophthalmus maximus*

	BAS	ATL	AD	BLS
BAS	—	**0.005**	**0.176**	**0.169**
ATL	**0.003/0.054**	—	**0.137**	**0.134**
AD	**0.089**/**0.217**	**0.055**/**0.057**	—	**0.213**
BLS	**0.072**/**0.131**	**0.035**/**0.271**	**0.074**/**0.222**	—

Above diagonal: the whole 755 SNPs dataset; below diagonal: 513 neutral/25 divergent outlier dataset; significance using 10,000 permutations, in bold face significant values after sequential Bonferroni correction (*p* < .008).

### 3.4 Identification of neutral and outlier loci

The three programs suggested a total of 234 outlier loci for divergent selection: BAYESCAN detected 10 outliers (*p *<* *95%; Figure S4), LOSITAN 127 (CI < 99%), and ARLEQUIN 190 (*p *<* *.050). When applying the most strict criteria (See Materials and Methods), 17 loci were identified (Table 4): 12 loci when evaluating the whole distribution range (global outliers), and smaller numbers when analyzing particular geographical regions (ATL (3), ATL & BAS (5), ATL & BLS (8), BAS & BLS (2), and BAS (4)).

**Table 4:** Loci under divergent or balancing selection in *Scophthalmus maximus* for the whole distribution (global) and for specific regions using three statistical approaches

Selection	SNP	Global	ATL	ATL & BAS	ATL&BLS	BAS & BLS	BAS‐N & BAS‐S
Divergent	11910_69	*/–/–					
	7193_56	**/–/–					
	16278_38		–/**/**				
	**1916_69**	**/**/**	**/**/**	**/*/**	**/–/**		
	1056_25	**/**/**		**/**/**			
	16775_23	*/**/**		–/**/**			
	2005_83	–/**/**			–/**/**		
	3865_39	*/**/**			*/*/**		
	5848_28	–/**/**			–/**/**		
	**5831_44**	–/**/**			–/**/**	**/**/**	
	**7574_88**	**/**/**			**/**/**	**/**/**	
	**6850_51**	**/**/**		**/**/**	**/**/**		
	**7550_55**	**/**/–	*/–/–	**/*/**	**/–/–		
	13736_17						–/**/**
	4628_55						–/**/**
	6478_39						–/**/**
	7733_27						–/**/**
Balancing	7033_88					–/*/**	
	5397_68					–/*/**	
	1587_12					–/*/**	
	2921_40					–/*/**	
	3659_32					–/*/**	
	7415_42					–/*/**	
	7698_82					–/*/**	
	7157_68						–/*/**

ATL, Atlantic; BAS, Baltic Sea; BLS, Black Sea; BAS‐N, north Baltic Sea; BAS‐S, south Baltic Sea.

Significance per locus is presented in the following order: BAYESCAN/ARLEQUIN/LOSITAN; asterisks indicate a posterior probability (*p*) of 95% (*) and 99% (**) for BAYESCAN; *p* < .05 (*) and 0.01 (**) for ARLEQUIN and a confidence interval of 99% (**) for LOSITAN; normal face: outliers detected in a single comparison; bold face: outliers detected in more than one comparison.

No outliers for stabilizing selection were detected by BAYESCAN, but 149 loci were detected with LOSITAN (140 outliers; *p* < .01) and ARLEQUIN (27 outliers; *p* < .05). Among these, eight outliers detected both with LOSITAN and ARLEQUIN, and consistent with environmental variation, were retained (Table 4). These outliers were mostly detected in BAS & BLS (7), two distant regions which share a low salinity environment, and an additional one was detected when comparing BAS populations (Table 4).

**Figure 4:**
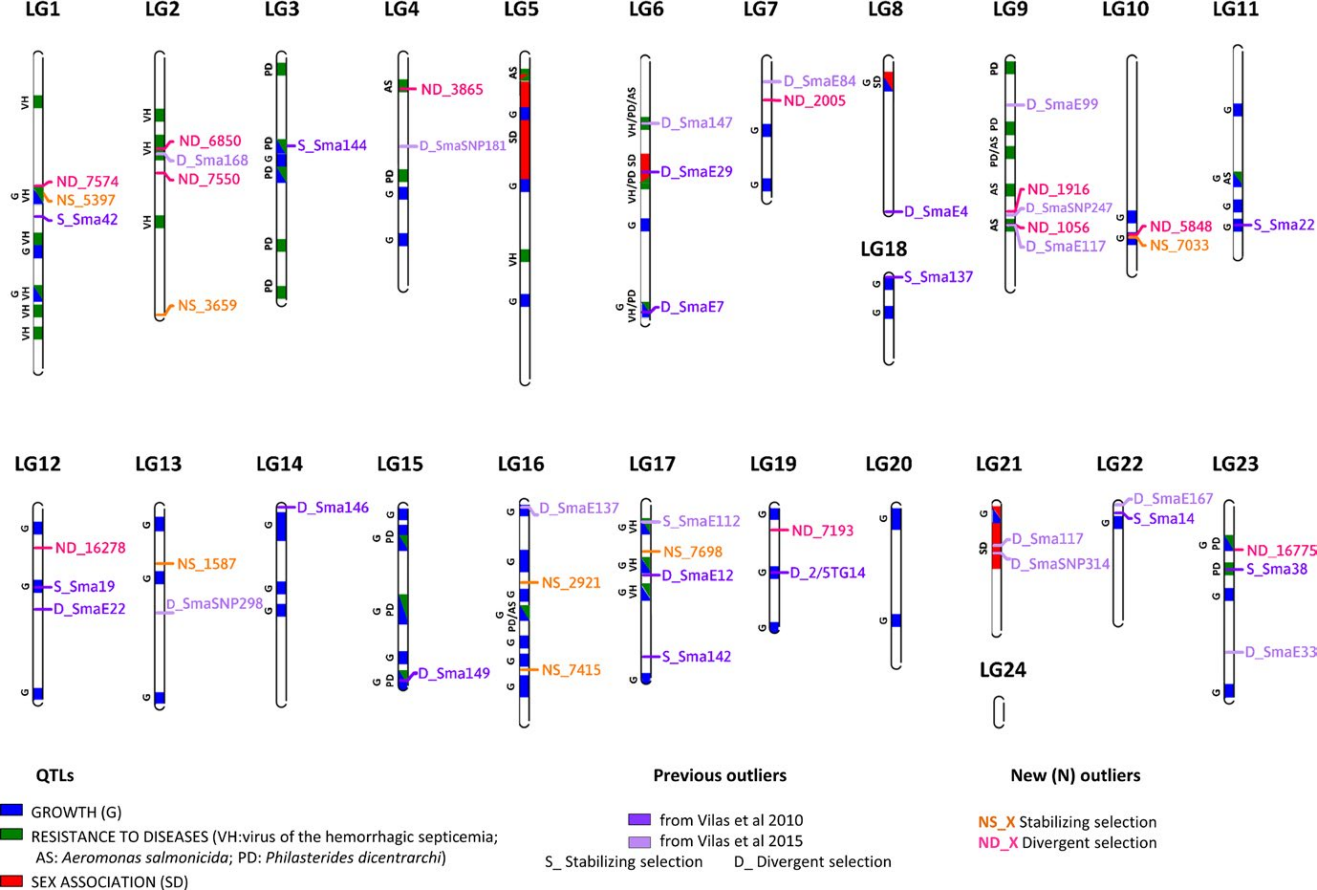
Linkage group position of outliers under divergent and stabilizing selection from this study and from Vilas et al. (2010, 2015) on *S. maximus* genetic map (LG: linkage groups) and their relation with previously reported QTLs. QTL labeling: (i) growth (BW: body weight; BL: body length; FK: Fulton's factor) in blue color, (ii) resistance to pathologies (AS:* Aeromonas salmonicida*; PD:* Philasterides dicentrarchi*; VHSV: viral hemorrhagic septicemia virus) in green color, and (iii) sex determination in red color

All divergent outliers (234) and the eight for balancing selection were removed from the dataset to obtain the most consistent set of 513 SNPs not showing evidence of selection, here designated as the “neutral panel.” Sequences of the 25 most consistent outlier loci (17 divergent + 8 balancing) were located in the turbot genome and most of them (23) anchored to the genetic map (Table S2, Figure 4). Three outlier loci co‐localized with previously reported outliers (6850_51, 1056_25 and 1916_69; Vilas et al., 2010, 2015) and six were found within the confidence intervals of QTL related to growth and resistance to pathologies, two traits of adaptive and productive relevance (Martínez et al., 2016; 7574_88, 5397_68, 6850_51, 1056_25, 5848_28, 7033_88; Table 5). Additionally, two pairs of outlier loci related to stabilizing and divergent selection, respectively (5397_68 and 7574_88 at LG1; 5848_28 and 7033_88 at LG10), were linked at <1 Mb distance. Overall, four relevant genomic regions at LG1, LG2, LG9, and LG10 were identified for further genome mining (±250 kb) and functional enrichment study (Table 5).

**Table 5:** Functional annotation and gene mining of the most relevant *Scophthalmus maximus* outliers

LG	SNP	Selection/Geographical region	Genomic region	Gene/Function	Gene mining	Functional enrichment	QTL and previous outliers
01	7574_88	Divergent Global ATL vs. BLS BAS vs. BLS	Intergenic	—	AQP1 (R/OS)	Metabolism	G^1^; VH^2^
01	5397_68	Stabilizing BAS vs. BLS	Intronic	ADGRL2 Exocytosis reg.	DHX30 (AS)		
02	6850_51	Divergent Global ATL vs. BAS ATL vs. BLS	Intronic	GPM6B Osteoblast differentation	VEGFC, KTR8, KTR18 (OS)	Metabolism and immune	VH^2^; V2015
09	1056_25	Divergent Global ATL vs. BAS	Intronic	MTMR7 Lipid and protein metabolism	NFIL3 (VH) ILF3, MICALL1, PATL1 (OS)	Metabolism and immune	AS^3^; V2015
09	1916_69	Divergent Global ATL ATL vs. BAS ATL vs. BLS	Intergenic				V2015
10	5848_28	Divergent Global ATL vs. BLS	Intronic	HDAC7 Macrophage differentiation	MTOR (G)	Metabolism and immune	G^1^
10	7033_88	Stabilizing BAS vs. BLS	Intronic	VSTM2L IG/G‐related	TWIST2 (OS)		

LG, linkage group; Selection: type of selection and geographical region; Gene/Function: official gene symbol and function (IG: immunoglobulin); data mining: relevant genes identified related to growth (G), VHSV resistance (VH), *Aeromonas salmonicida* resistance (AS), reproduction (R) and osmoregulation (OS); QTLs and previous outliers from ^1^Sánchez‐Molano et al., 2011; ^2^Rodríguez‐Ramilo et al., 2014; ^3^Rodríguez‐Ramilo et al., 2011; V2015: outliers reported by Vilas et al., 2015.

LG1: Outlier 5397_68 corresponded with an intronic region of *ADGRL2* (adhesion G protein‐coupled receptor L2), a gene related to exocytosis regulation (Ushkaryov, Volynski, & Ashton, 2004), while 7574_88 was located in an intergenic region. This latter outlier showed a gradual cline in allele frequency from the northernmost samples, through the Mediterranean, and up to the Black Sea: the most common allele in the Baltic (0.879) showed a range between 0.938 (ICE; North Atlantic) and 0.650 (BB‐FR; Biscay Bay) in ATL populations, a frequency of 0.382 in the single Mediterranean population (AD), and the lowest frequency in BLS (0.019‐ 0.029). The two aforementioned outlier loci were located within the confidence intervals of two overlapping QTLs for resistance to the hemorrhagic septicemia virus (VHSV) and growth (Rodríguez‐Ramilo et al., 2014) and closely linked to the important growth‐related gene *IGFBP3* (insulin like growth factor binding protein 3; Robledo et al., 2016). Data mining around 5397_68 identified a candidate gene related to *Aeromonas salmonicida* resistance, *DHX30* (DExH‐box helicase 30; Figueras et al., 2016), while mining around 7574_88 identified *AQP1* (aquaporin 1), a gene related to fertilization (Figueras et al., 2016), and osmoregulation (Grady, Knepper, Burg, & Ferraris, 2014). Functional enrichment in the vicinity of these markers identified metabolic processes (Table 5).

LG2: Outlier 6850_51 mapped to an intronic region of *GPM6B* (Glycoprotein M6B), a gene strongly up‐regulated during osteoblast differentiation in humans (Drabek, van de Peppel, Eijken, & van Leeuwen, 2011). This outlier is within the confidence interval of a VHSV resistance QTL and relatively close to a previously reported outlier (~3 cM; Sma‐USC168; Vilas et al., 2015). Data mining identified candidate genes related to osmoregulation (*VEGFC* (vascular endothelial growth factor C), *KRT8* (keratin 8), *KRT18* (keratin18); Grady et al., 2014). Functional enrichment showed genes related to metabolism and immunity (Table 5).

LG9: Outlier 1056_25 was located within an intron of *MTMR7* (myotubularin‐related protein 7), a gene related to lipid and protein metabolism (Safran et al., 2010). This gene is within the confidence interval of an *A*.* salmonicida* resistance QTL and tightly linked to a previously reported outlier (~ 63 kb; Sma‐E117; Vilas et al., 2015). Data mining revealed the presence of *NFIL3* (nuclear factor, interleukin 3 regulated), a candidate gene for VHSV resistance (Figueras et al., 2016) and three candidate genes related to osmoregulation *ILF3* (interleukin enhancer binding factor 3), *MICALL1* (MICAL like 1), and *PATL1* (PAT1 homolog 1, processing body mRNA decay factor; Grady et al., 2014). The outlier 1916_69 was located in an intergenic region, relatively close to a previously reported SNP outlier (<3 cM; SmaSNP247; Vilas et al., 2015). Functional enrichment around both outlier loci showed genes related to metabolism and immunity (Table 5).

LG10: Outlier 5848_28 was located within an intron of *HDAC7* (histone deacetylase 7), a gene which plays an important role in macrophage differentiation (Das Gupta, Shakespear, Iyer, Fairlie, & Sweet, 2016), while 7033_88 was within an intron of *VSTM2L* (V‐set and transmembrane domain containing 2 like), a poorly characterized gene with conserved immunoglobulin‐like domains, related to neuronal modulation and associated with growth traits in cattle (Espigolan et al., 2015). Both outlier loci were within the confidence interval of a growth‐related QTL (Sánchez‐Molano et al., 2011) and data mining detected the presence of *MTOR* (mechanistic target of rapamycin), a candidate gene related to growth and *A*.* salmonicida* resistance (Figueras et al., 2016), and *TWIST2* (twist family BHLH transcription factor 2), a gene related to osmoregulation (Grady et al., 2014). Functional enrichment displayed GO terms related to primary metabolism and immunity (Table 5).

### 3.5 Seascape analysis

**Figure 5:**
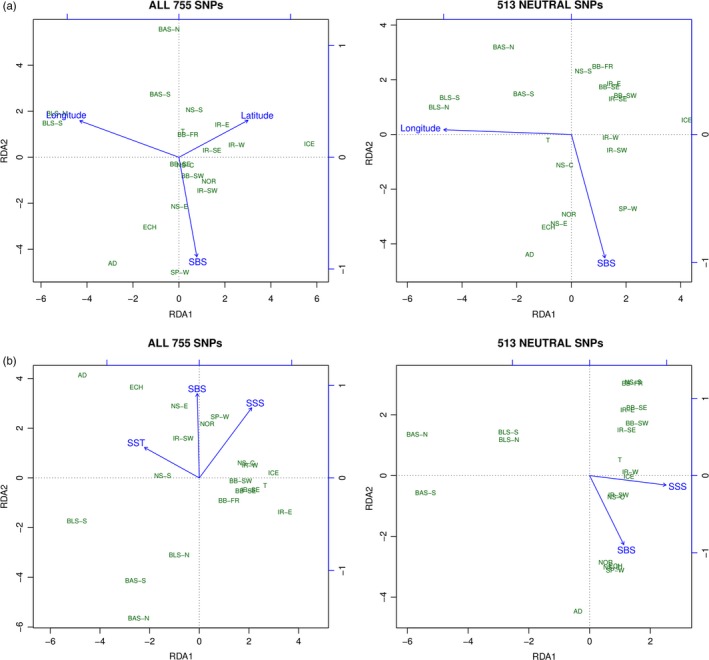
Redundancy analyses (RDA) of *Scophthalmus maximus* samples originating from the entire distribution area using the complete 755 SNP panel and the 513 neutral SNPs. In (a), using a forward selection model starting from all landscape variables (Model 1) and (b) only from environmental variables (Model 2)

All environmental variables showed VIF values below 5, suggesting no collinearity issues between them. Redundancy analyses including spatial and environmental variables (Table 1) and the full genetic dataset suggested latitude, longitude, and sea bottom salinity (SBS) as putative drivers for genetic differentiation across the total study area (Table 6, Figure 5a). Latitude and longitude were associated with the first axis, while SBS was associated with the second axis of the RDA plot. Using the neutral dataset, latitude was not significant. When only environmental variables and the full dataset were included (Figure 5b), sea surface temperature (SST), sea surface salinity (SSS), and SBS were significant, with SBS mostly associated with the first axis and SST associated with the second one. The four main turbot regions (ATL, BAS, AD, and BLS) were roughly separated in this analysis: BAS and BL were associated with low salinity; AD with high temperature; and the Atlantic group with high surface salinity, but showing a high variability in surface temperature and less in sea bottom salinity. Finally, only marginal significance was found for SSS and SBS when the neutral dataset was used.

**Table 6:** Results of the redundancy analysis (RDA) on *Scophthalmus maximus* populations

Model	Environmental variable	All markers		Neutral markers	
		*p*‐Value	Adjusted *R* ^2^	*p*‐Value	Adjusted *R* ^2^
Model 1	Longitude	.001	.141	.002	.078
	Latitude	.017		—	
	SBS	.025		.047	
Model 2	SST	.034	.106	—	.049
	SSS	.010		.055	
	SBS	.041		.053	

Model 1: Forward selection model starting from all landscape variables; Model 2: only from environmental variables. Environmental codes are shown in Table 1.

### 3.6 Genetic structure based on neutral and outlier markers

Population structure was reanalyzed using the most consistent neutral (513 loci) and outlier (25 loci) SNP datasets to compare patterns of genetic differentiation related to demographic parameters and selection (Table 4). A third subpanel was also used to analyze differentiation in the ATL and BAS using the six outlier loci detected in these comparisons (16278_38, 1916_69, 1056_25, 16775_23, 6850_51, and 7550_55; Table 4).

Levels of genetic differentiation estimated for the neutral panel were lower than estimated from the full SNP dataset (Table 3, Figures S2b and S5), but remained statistically significant between ATL versus BAS (*F*_ST_ = 0.003, *p *<* *.050), ATL versus BLS (*F*_ST_ = 0.035, *p *<* *.001), and BAS versus BLS (*F*_ST_ = 0.072, *p* < .0001). The global differentiation pattern was similar to that observed when using the whole marker dataset (Figures S1 vs. S5, respectively), with samples being split in the four main geographical regions (ATL, BAS, AD, and BLS). Interestingly, the DAPC analyses showed similarity between BAS‐N and ATL (Figure S2b). N_e_ was also recalculated for all sites using the neutral dataset, but similar results were obtained (data not shown).

When only ATL samples were examined, DAPC with neutral markers yielded similar results as the full panel indicating that turbot from Norway and Spanish coasts were slightly differentiated from the other samples (Figure S2c). Nevertheless, no isolation by distance (IBD) was apparent in the region (*r *=* *.07648, *p *=* *.297).

Based solely on the 25 outlier SNPs, *F*_ST_ markedly increased between ATL versus BAS (*F*_ST_ = 0.054; *p *<* *.0001) and ATL versus BLS (*F*_ST_ = 0.271; *p *<* *.0001; Table 3). *F*_ST_ was also high between BAS versus BLS (*F*_ST_ = 0.131, *p *<* *.0001), but lower than that computed from the full SNP panel (*F*_ST_ = 0.169), suggestive of stabilizing selection acting on some of these outlier loci in both regions. Genetic structure resolved by only the six ATL‐BAS divergent outlier loci showed that both BAS and NOR were distinct from the remaining ATL samples, as three main clusters were identified both with STRUCTURE (Figure S6) and DAPC (Figure S2d) analyses.

## 4 DISCUSSION

### 4.1 Genetic diversity and population structure

In this study, the population genetic structure of turbot was analyzed for the first time using a genomewide SNP panel (>1 SNP per Mb) developed with a genotyping‐by‐sequencing (GBS) strategy, namely ddRAD (Peterson et al., 2012). This allowed us to screen populations from the entire distribution range avoiding the ascertainment bias that can affect analyses with fixed SNP panels (e.g., SNP chips) developed from only a few populations. The combination of balanced multiplexing of individuals per library along with the progressive lowering cost of high‐throughput sequencing made the approach cost‐effective. A set of 755 highly confident SNPs (0.5% genotyping error) were obtained covering homogeneously the turbot genome at >1 SNP/Mb from the initial 5,564 SNPs. The proportion of filtered SNPs was in line with those previously reported in marine fish species (Palaiokostas et al., 2015) and the genotyping error was even lower (Mastretta‐Yanes et al., 2015), supporting the robustness of our custom filtering pipeline.

Our analysis represents the largest effort to date to analyze the genetic diversity and structure of turbot across its entire distribution range including the Atlantic Ocean and the Mediterranean Sea, as well as the inner Baltic and Black seas. Our sample collection was rather uneven due to the overrepresentation of the Atlantic area (14 of 20 samples), which suggests some caution for statistical bias when analyzing the whole dataset. However, this sampling reflects quite well turbot distribution, which is common in the Atlantic Ocean and very scarce in the Mediterranean Sea, and therefore, the analysis should take this fact into consideration. Anyway, we split our analysis also by region and made all meaningful comparisons between regions to obtain the most comprehensive view of turbot structure.

Genetic diversity in the Atlantic Ocean (H_E_: 0.095) was much lower than previously reported (Vilas et al., 2015; H_E_: ~0.300), mainly due to the high number of samples used to identify polymorphic loci in our study (697) and the low MAF cutoff used (0.002). This resulted in the detection of a large fraction of loci with very rare alleles compared to previous studies (sample size ~30; Vera et al., 2013; Vilas et al., 2015). In fact, genetic diversity estimated using the fraction of loci at P_95_ almost doubled (H_E_: 0.184) approaching to previous results (Vilas et al., 2015). Anyway, our data suggest that genetic diversity of turbot features in the lower range of values reported for other marine fish studied with SNP panels, such as European sea bass *Dicentrarchus labrax* (from 0.233 in the Atlantic Ocean to 0.290 in the West Mediterranean Sea; Souche et al., 2015), Atlantic herring (*Clupea harengus*; 0.270–0.310 along the Atlantic Ocean and the Baltic Sea; Limborg et al., 2012), and Asian sea bass (*Lates calcarifer*; 0.292–0.411; Wang, Wan, Lim, & Yue, 2015).

Given the general lack of physical barriers in the sea, marine fish, such as turbot, with wide distribution ranges, high fecundity, pelagic eggs, and larvae, are expected to be at best weakly structured across large areas. The only study exploring genetic structure in turbot covering a roughly similar distribution range was based on allozyme marker data (Blanquer et al., 1992). The reported global population differentiation (*F*_ST_ = 0.070) was similar to the current study (*F*_ST_ = 0.090), especially considering that the allozyme study did not include the Black Sea. In our work, turbot samples were consistently separated into four main genetic regions: Atlantic, Baltic Sea, Black Sea, and Adriatic Sea. However, it should be kept in mind that turbot is essentially a northeastern Atlantic species with relict populations in the Mediterranean Sea, such as in the Gulf of Lion and the northern Adriatic Sea. Its isolated occurrence in the Black Sea can be attributed to the more suitable environmental conditions compared to the Mediterranean Sea for a temperate cold‐water species. Hence, genetic differentiation between the sampled Atlantic and Baltic turbot was much lower (*F*_ST_ = 0.007) than between the Atlantic and either Adriatic (*F*_ST_ = 0.170) or Black Sea (*F*_ST_ = 0.240) samples.

Very low genetic differentiation between samples was observed within the Atlantic region, supporting previous findings (Bouza et al., 2002; Coughlan et al., 1998; Florin & Höglund, 2007; Nielsen et al., 2004; Vandamme et al., 2014; Vilas et al., 2015), which indicates relatively high levels of gene flow. This is the case notwithstanding the presence of different well‐known current fronts inside the large marine ecosystems (LME) surveyed in this study, such as the Iberian Coastal, Irish and Biscay Shelf, and North Sea (Belkin & Cornillon, 2007; Belkin, Cornillon, & Sherman, 2009; Vandamme et al., 2014). Within this relatively homogeneous genetic scenario, subtle, but rather consistent differences along with low N_e_ estimates were detected at both geographical extremes, that is, Norwegian and Spanish Coast samples. Vilas et al. (2015) also suggested that Iberian turbot should be considered genetically distinct from elsewhere in the Atlantic region. Restricted gene flow to more northern regions due to oceanic fronts off the Galician coast has been suggested for other species with passive larval dispersal such as the flat oyster *Ostrea edulis* (Vera et al., 2016a).

Low but significant genetic differentiation was detected between the Atlantic and Baltic Sea turbot (*F*_ST_ = 0.005; *p *<* *.001), a substructure previously reported based on other SNP and microsatellite genotypes (Nielsen et al., 2004; Vandamme et al., 2014; Vilas et al., 2010, 2015) and, in part, attributed to the biogeographic barrier occurring between the North Sea and the Baltic Sea (Johannesson & André, 2006). Marginal or geographically isolated populations are often more prone to the effects of genetic drift and show higher genetic divergence and lower diversity than those closer to the center of the species distribution (Kawecki, 2008; Lira‐Noriega & Manthey, 2014). Other marine fish distributed in the Baltic Sea also show lower genetic diversity than conspecific Atlantic populations due to varying processes of isolation over 4,000–8,000 years since colonization after the last glaciation (Littorina period; Johannesson & André, 2006). The more pronounced deviations from HWE observed in the Baltic samples may suggest a Wahlund effect, the Baltic Sea being a more heterogeneous environment and having a relatively recent history of colonization from the Atlantic, constituting a partial transitional environment (Nielsen et al., 2004).

### 4.2 Local adaptation in turbot

The detection of signals for divergent selection in genomes is favored in scenarios of relatively large *N*_*e*_, as in turbot, because the genetic signal left by the demographic history will be easier to erode and the ability to detect high differentiation outliers is favored by a low baseline level of neutral genetic differentiation between populations. Although turbot populations generally exhibited low to moderate genetic structure, populations from the Black Sea and the Adriatic Sea showed evidence of geographical isolation. The discrimination between neutral and selective variation in the Baltic‐Atlantic transition zone may be further complicated as correlations between genetic and environmental variation might be due to reasons other than natural selection (Bierne, Roze, & Welch, 2013; Bierne et al., 2013). Therefore, outlier loci were carefully assessed in our study and were only considered reliable when they showed strong statistical support and previous phenotypic information consistent with predefined hypotheses. Genomic co‐localization with previously reported outlier loci or association with growth or disease‐resistance‐related QTL evaluated so far in turbot (Martínez et al., 2016) was also considered, as they might point out to genomic islands of divergence, as reported in other fish (Bradbury et al., 2013).

Evidence of divergent selection in turbot was mainly detected in the comparisons between the Atlantic region and those regions with low salinity (BAS and BLS). Differentiation due to selection (and genetic drift) may be favored by limited gene flow related to differences in salinity tolerance. It is known that Atlantic turbot eggs do not survive at the lower salinities of the Baltic Sea (Florin & Höglund, 2007), and, in addition, because turbot eggs are not buoyant at salinities below 20 PSU, eggs from the Baltic are demersal rather than pelagic (Nissling et al., 2006). Three of the five markers that were statistically significant in the ATL‐BAS comparison (1916_69 at LG9, 6850_51 and 7550_55 at LG2) were also significant in the comparison between ATL and BLS, strongly supporting that their divergence might be related to adaptation to differences in salinity. Furthermore, the marker 1916_69 is closely linked to a previously reported outlier (*SmaSNP247*; Vilas et al., 2015), and both of these markers show a pattern consistent with divergent selection and are located relatively close (3 cM distant; Figueras et al., 2016). Although 1056_25, also at LG9, was only divergent in the comparison with BAS, it was located within an important functional region including genes related to osmoregulation and is tightly linked to a previously reported divergent outlier (*SmaE117*) between the Atlantic Ocean and the Baltic Sea (Vilas et al., 2015). These results suggest that several loci within a narrow region at LG9 show a spatial pattern of structuring consistent with adaptation to environments that differ markedly in salinity.

Marker 7574_88 selectively diverged in the ATL‐BLS comparison with all three analytical tests employed, but not in the ATL‐BAS comparison. Most important, this outlier is located in a genomic region related to growth (Figueras et al., 2016; Rodríguez‐Ramilo et al., 2014), and further, it showed a gradual cline from Baltic through to Black Sea samples. A similar pattern was detected for 2372_27, a linked marker at LG1 (~400 kb distant from 7574_88), which was significant for divergent selection with LOSITAN and ARLEQUIN. These differences might be related to temperature variation following a north–south cline facilitating adaptation through growth‐related loci (Nissling, Florin, Thorsen, & Bergström, 2013; Vilas et al., 2015).

Interestingly, the outlier 5397_68, located very close to 2372_27 at LG1 (<100 kb distant), showed signals of stabilizing selection in the BAS‐BLS comparison. The marker SNP 5397_68 is located in the vicinity (~100–400 kb distant) of genes related to growth (e.g., *GPAA1*,* EXOSC4*,* PRKACB*), and further, Norman, Ferguson, and Danzmann (2014) detected several QTLs related to salinity tolerance in an orthologous region in Arctic charr (*Salvelinus alpinus*, LG32), which contains three genes related to osmoregulation (*EFID*,* PPM1L* and *UCK2*). This genomic region at LG1, which includes several outlier loci, growth and VHS resistance QTLs, and growth and salinity tolerance candidate genes, seems relevant to explain the genetic structure of turbot.

Although still a controversial topic, balancing selection (or parallel evolution) has been identified as an important factor in the evolution of some marine species, for example, related to coral reef fishes (Gaither et al., 2015), European sea bass (Lemaire et al., 2000), and three‐spined stickleback (Feulner et al., 2013; Guo, DeFaveri, Sotelo, Nair, & Merilä, 2015). Moreover, it has also been suggested to play an important role in invasive processes of aquatic organisms (Vera, Díez‐del‐Molino, & García‐Marín, 2016b). Adaptive variants maintained by balancing selection have been reported in different species, such as those in immune‐related genes in three‐spined stickleback (Feulner et al., 2013; Guo et al., 2015) and in ribosomal structure and regulation genes in the albacore tuna (Laconcha et al., 2015). Seven of the eight outlier loci identified as being influenced by balancing selection in the current study were detected in the BAS‐BLS comparison, a result consistent with parallel adaptation to low salinities. As expected, *F*_ST_ between BAS and BLS increased when these outliers were excluded (using only neutral markers). As mentioned above, two of these markers (7033_88 and 5397_68) are linked to several growth‐related loci (Robledo et al., 2016; Rodríguez‐Ramilo et al., 2014; Sánchez‐Molano et al., 2011), and also to some candidate genes associated with osmotic stress response (Norman et al., 2014; Ortells et al., 2012). Two other outlier loci reside close to genes related to osmoregulation: 1587_12 on LG13 near *RAC1* and *PRKCA* (Di Ciano et al., 2002; Zhuang, Hirai, & Ohno, 2000), and 2921_40 on LG16 near *NAP1L1* (Wang et al., 2014).

In summary, our data not only confirm previous genetic diversity and population structure data based on different markers, but reveals crucial novel information on population adaptation and connectivity using a combination of neutral and adaptive genetic variation. Low but significant differentiation was detected between the Atlantic and Baltic regions, while high differentiation was observed between the Atlantic and the southeastern‐most regions (Adriatic and Black seas), indicating that demographic and historical factors have contributed to shaping population structure of turbot across its natural distribution. The information reported here also provides for the first time new insights on turbot adaptation, especially in the Black Sea, and suggested parallel evolution between areas with similar environmental conditions. Both strong neutral evolutionary forces and adaptive selection appear to be acting simultaneously on geographically isolated populations in the natural distribution of turbot. However, subtle neutral differentiation and local adaptations might also be occurring within regions. Candidate outlier loci, mostly anchored to the turbot genome, and especially at specific regions of LG1 and LG9, showed a positive correlation with environmental variables related to salinity and temperature.

### 4.3 Management implications

Our results represent useful information for the management of wild stocks, and they can be valuable for breeding programs of farmed turbot. An improved definition of management units considering both demography and adaptation to environmental variation along the whole distribution range can now be delineated, allowing the future definition of adaptive management units (AMU, Bernatchez et al., 2017). Four main operational units can be defined related to the four main genetic regions identified along the distribution range, but further refinement should be considered within the Atlantic area, where both Norway and Spanish samples showed slight differentiation from the Atlantic core both using all data and outlier loci. Our study did not detect significant subdivision in the British Isles and the North Sea as previously suggested by Vandamme et al. (2014). Further, although only two samples were analyzed, the North and South Baltic Sea showed significant differences, both when considering the full dataset, and outlier and neutral loci separately. In addition, our data represent the baseline to monitor restocking performed in the Atlantic area with unknown consequences, and when the genetic composition of the broodstock of the main turbot farms be at hand, to evaluate the impact and introgression from farm escapes to evolve toward a sustainable aquaculture. Finally, providing breeders with information of natural resources regarding environmental variables will be highly useful to boost breeding programs fitting them to market demands, to found broodstock at farms in new geographical regions, and to face the new challenges of climatic change.

## CONFLICT OF INTEREST

None declared.
