# Supplementary material for: Parallel evolution and adaptation to environmental factors in a marine flatfish: Implications for fisheries and aquaculture management of the turbot (Scophthalmus maximus)
Source: Evol Appl. 2018 Apr 6;11(8):1322–41. doi: 10.1111/eva.12628 (PMC6099829; doi:10.1111/eva.12628)
Supplement: Supplementary file 1 [file EVA-11-1322-s001.docx]

**Supporting information**

**Table S1**. Pairwise F_ST_ matrix of all *S*. *maximus* samples genotyped for the full 755 SNP panel.

|  | *BAS-N* | *BAS-S* | *T* | *NOR* | *NS-E* | *NS-C* | *NS-S* | *ICE* | *IR-W* | *IR-E* | *IR-SW* | *IR-SE* | *ECH* | *BB-FR* | *BB-SE* | *BB-SW* | *SP-W* | *AD* | *BLS-N* | *BLS-S* |
| --- | --- | --- | --- | --- | --- | --- | --- | --- | --- | --- | --- | --- | --- | --- | --- | --- | --- | --- | --- | --- |
| *BAS-N* | - |  |  |  |  |  |  |  |  |  |  |  |  |  |  |  |  |  |  |  |
| *BA-S* | 0.001 | - |  |  |  |  |  |  |  |  |  |  |  |  |  |  |  |  |  |  |
| *T* | 0.006 | 0.000 | - |  |  |  |  |  |  |  |  |  |  |  |  |  |  |  |  |  |
| *NOR* | **0.021** | 0.007 | 0.003 | - |  |  |  |  |  |  |  |  |  |  |  |  |  |  |  |  |
| *NS-E* | **0.008** | 0.000 | 0.000 | 0.003 | - |  |  |  |  |  |  |  |  |  |  |  |  |  |  |  |
| *NS-C* | **0.011** | 0.000 | 0.000 | 0.003 | 0.000 | - |  |  |  |  |  |  |  |  |  |  |  |  |  |  |
| *NS-S* | **0.016** | 0.004 | 0.000 | 0.002 | 0.000 | 0.000 | - |  |  |  |  |  |  |  |  |  |  |  |  |  |
| *ICE* | 0.013 | 0.000 | 0.003 | 0.000 | 0.000 | 0.000 | 0.000 | - |  |  |  |  |  |  |  |  |  |  |  |  |
| *IR-W* | **0.017** | 0.001 | 0.000 | 0.000 | 0.000 | 0.000 | 0.000 | 0.000 | - |  |  |  |  |  |  |  |  |  |  |  |
| *IR-E* | **0.019** | **0.005** | 0.000 | 0.000 | 0.000 | 0.000 | 0.000 | 0.000 | 0.000 | - |  |  |  |  |  |  |  |  |  |  |
| *IR-SW* | 0.008 | 0.000 | 0.000 | 0.004 | 0.004 | 0.006 | 0.004 | 0.000 | 0.005 | 0.005 | - |  |  |  |  |  |  |  |  |  |
| *IR-SE* | 0.010 | 0.000 | 0.000 | 0.001 | 0.000 | 0.000 | 0.000 | 0.000 | 0.001 | 0.003 | 0.005 | - |  |  |  |  |  |  |  |  |
| *ECH* | 0.010 | 0.005 | 0.000 | 0.009 | 0.006 | 0.004 | 0.000 | 0.000 | 0.005 | 0.002 | 0.006 | 0.000 | - |  |  |  |  |  |  |  |
| *BB-FR* | **0.010** | 0.000 | 0.000 | 0.007 | 0.000 | 0.000 | 0.000 | 0.000 | 0.000 | 0.000 | 0.003 | 0.000 | 0.000 | - |  |  |  |  |  |  |
| *BB-SE* | **0.013** | **0.006** | 0.000 | 0.008 | 0.000 | 0.000 | 0.000 | 0.000 | 0.000 | 0.000 | 0.006 | 0.000 | 0.000 | 0.000 | - |  |  |  |  |  |
| *BB-SW* | **0.023** | **0.007** | 0.000 | **0.012** | 0.004 | 0.005 | 0.002 | 0.000 | 0.000 | 0.004 | 0.007 | 0.005 | 0.005 | 0.000 | 0.000 | - |  |  |  |  |
| *SP-W* | **0.015** | 0.000 | 0.000 | 0.005 | **0.009** | **0.007** | 0.002 | 0.000 | 0.003 | **0.005** | **0.010** | 0.006 | **0.008** | **0.006** | 0.003 | **0.006** | - |  |  |  |
| *AD* | **0.163** | **0.174** | **0.157** | **0.148** | **0.130** | **0.138** | **0.131** | **0.139** | **0.130** | **0.138** | **0.148** | **0.124** | **0.140** | **0.145** | **0.133** | **0.132** | **0.144** | - |  |  |
| *BLS-N* | **0.160** | **0.156** | **0.138** | **0.120** | **0.127** | **0.111** | **0.133** | **0.132** | **0.113** | **0.127** | **0.128** | **0.107** | **0.123** | **0.131** | **0.135** | **0.132** | **0.126** | **0.207** | - |  |
| *BLS-S* | **0.167** | **0.176** | **0.147** | **0.140** | **0.147** | **0.132** | **0.144** | **0.141** | **0.133** | **0.142** | **0.147** | **0.125** | **0.137** | **0.145** | **0.149** | **0.150** | **0.150** | **0.207** | 0.000 | - |

Significance after 10.000 permutations; bold: P < 0.05; underlined and bold: significant values after sequential Bonferroni correction (P < 0.0002).

**Figure S1.** STRUCTURE results for all wild *S*. *maximus* genotyped for the full 755 SNP panel. Results are shown from K=2 to 5 genetic clusters.


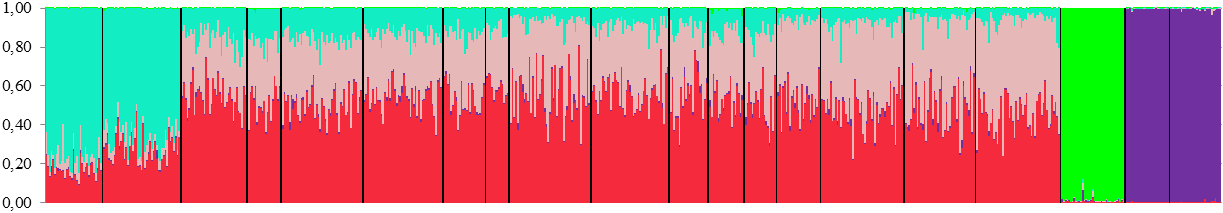

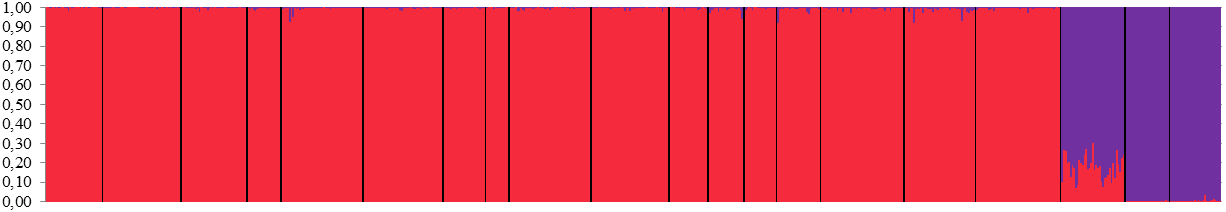


BAS-N BAS-S T NOR NS-E NS-C NS-S ICE IR-W IR-E IR-SW IR-SE ECH BB-FR BB-SE BB-SW SP-W AD BLS-N BLS-S


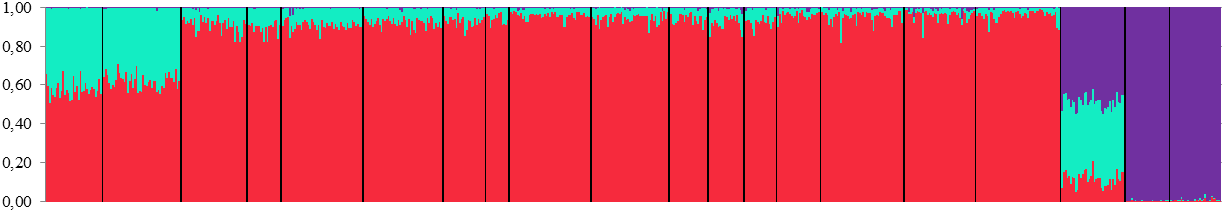

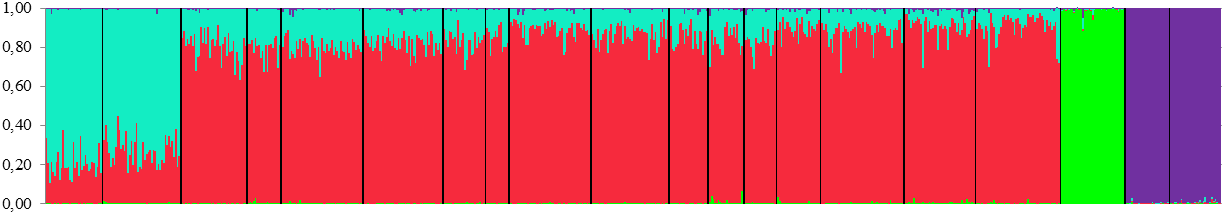


**Figure S2.** DAPC of all *S*. *maximus* (a, b), Atlantic (c) and Atlantic & Baltic (d) samples. Samples were genotyped for the 755 full SNP panel (a), the 513 neutral SNPs (b, c) and the six divergent outliers involving the Atlantic and Baltic Sea (d).


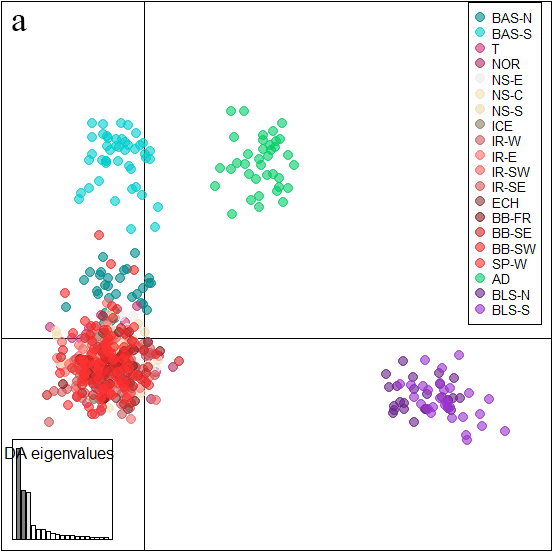

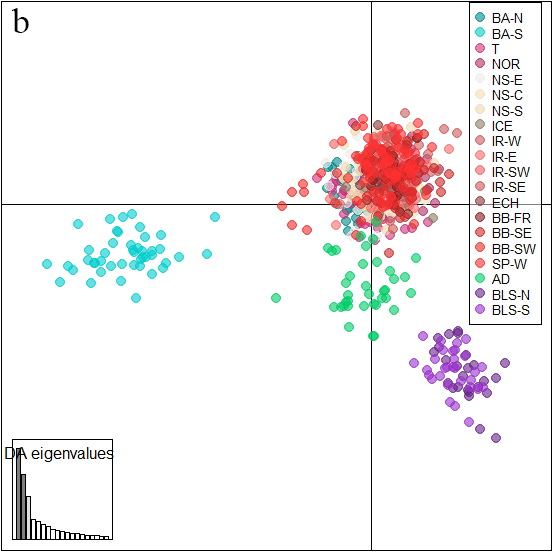

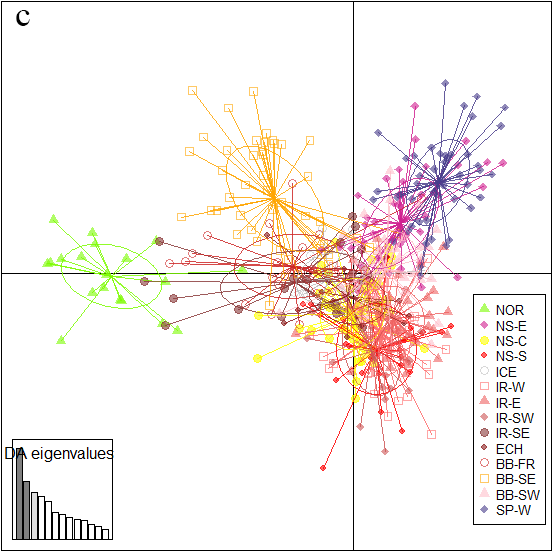

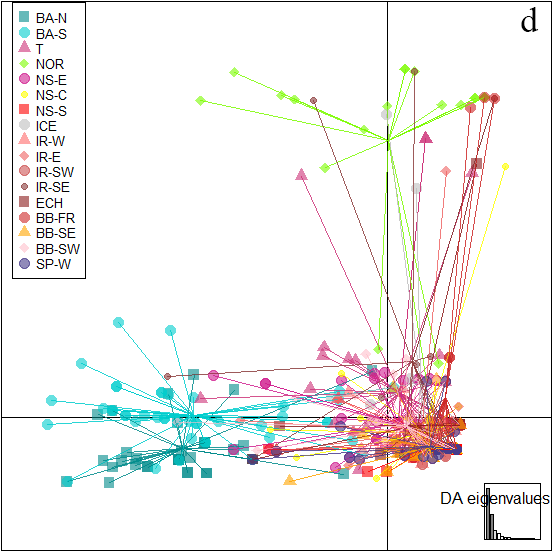


**Figure S3.** STRUCTURE results for Atlantic *S*. *maximus* genotyped for the full 755 SNP panel. Results are shown from K=2 to 4 genetic clusters.


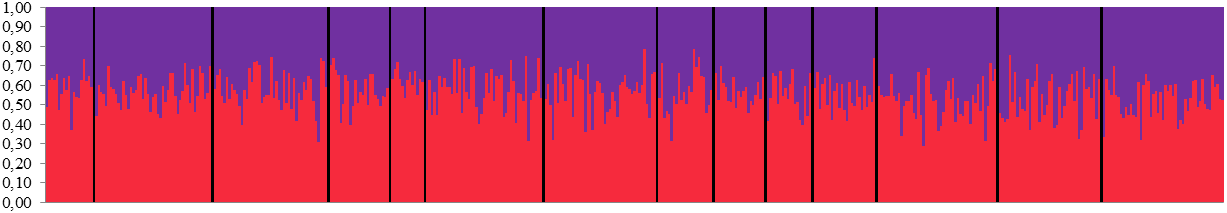


NOR NS-E NS-C NS-S ICE IR-W IR-E IR-SW IR-SE ECH BB-FR BB-SE BB-SW SP-W


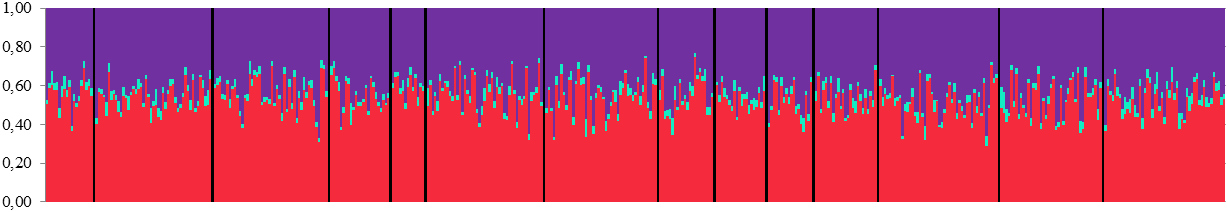

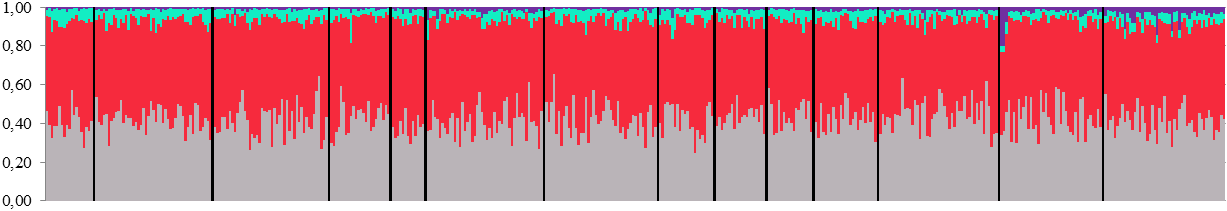


**Figure S4.** Graphical representation of *S*. *maximus* outliers for divergent selection detected with Bayescan. Filled black circles represent candidate outlier SNPs. Continuous and dashed vertical lines show P = 0.99 (Log10(BF) > 2) and P = 0.95 (Log10(BF) > 1.3) thresholds. Global: all the 20 samples (a); Atlantic (b); Atlantic & Baltic Sea (c); Atlantic & Black Sea (d): Baltic & Black Sea (e).


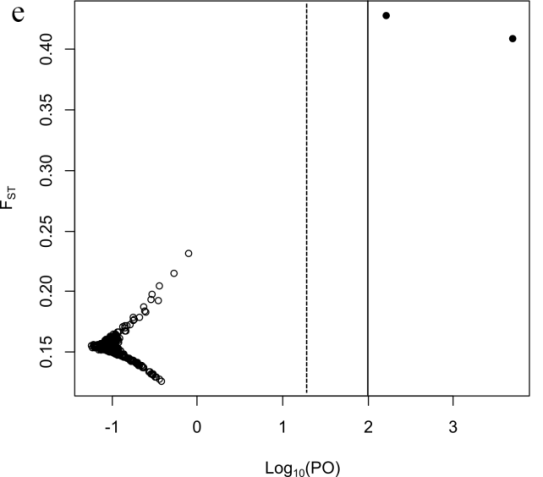

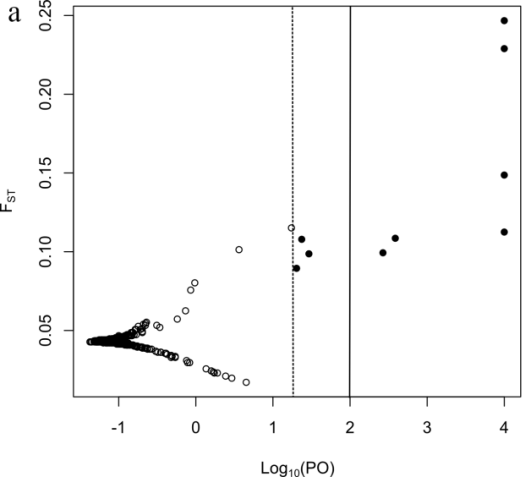

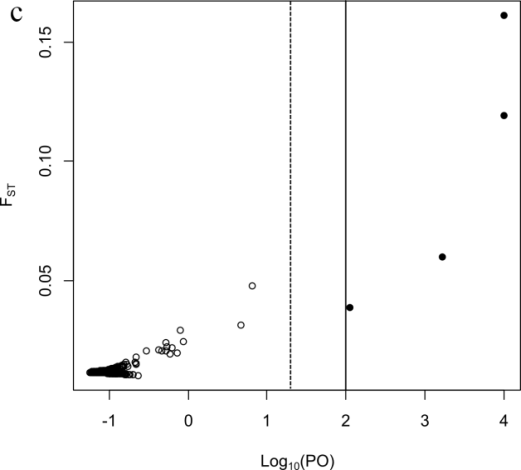

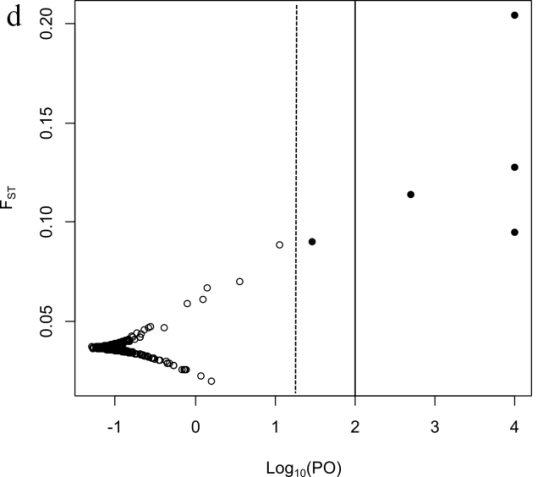

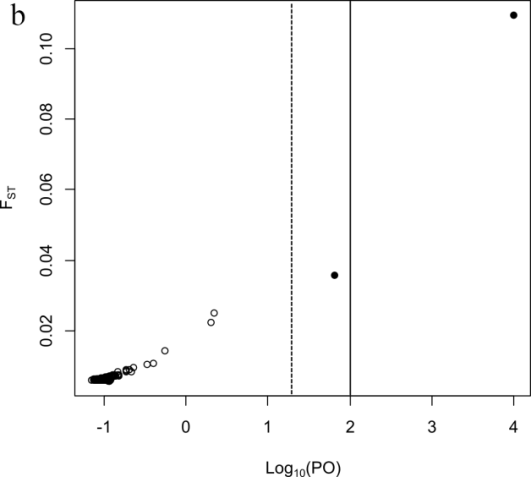


**Figure S5** STRUCTURE results for all wild *S*. *maximus* genotyped at the 513 neutral SNP dataset. Results are shown from K = 2 to 5 genetic clusters.

BAS-N BAS-S T NOR NS-E NS-C NS-S ICE IR-W IR-E IR-SW IR-SE ECH BB-FR BB-SE BB-SW SP-W AD BLS-N BLS-S


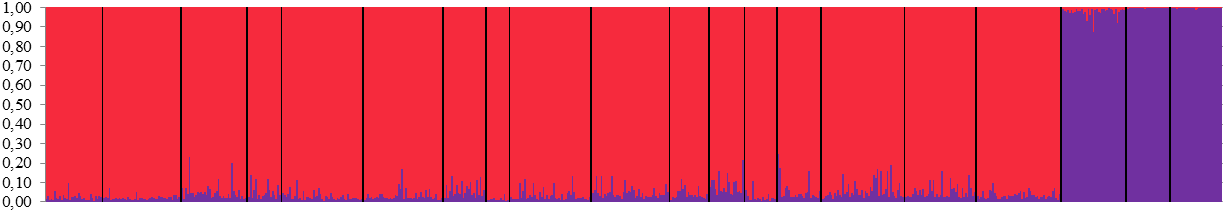

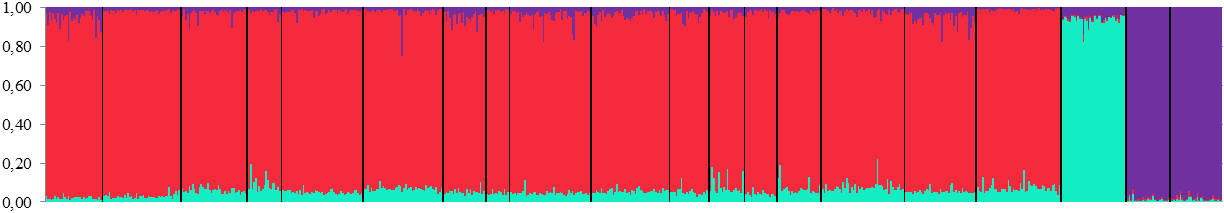

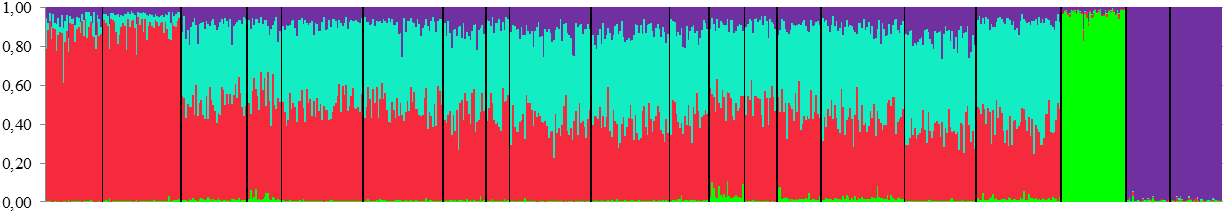

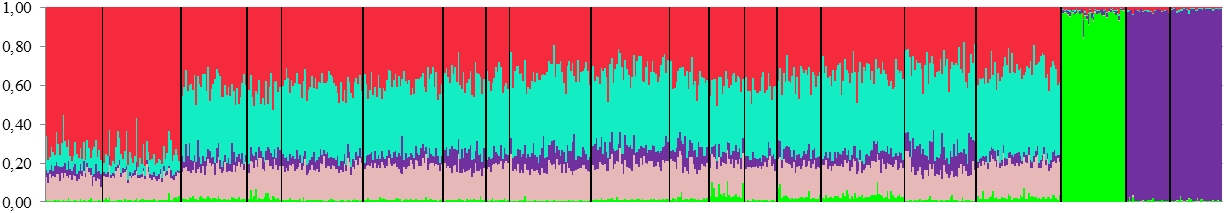


**Figure S6.** STRUCTURE results for Atlantic and Baltic *S*. *maximus* genotyped at the six divergent outliers in the area. Results are shown from K=2 to 5 genetic clusters.


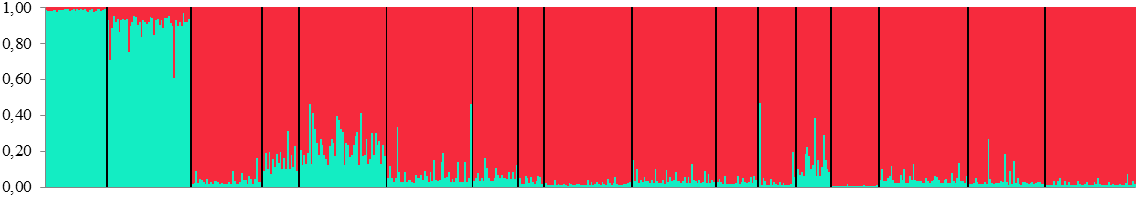

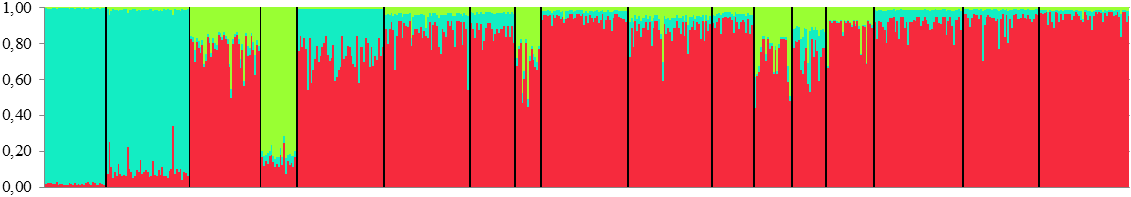

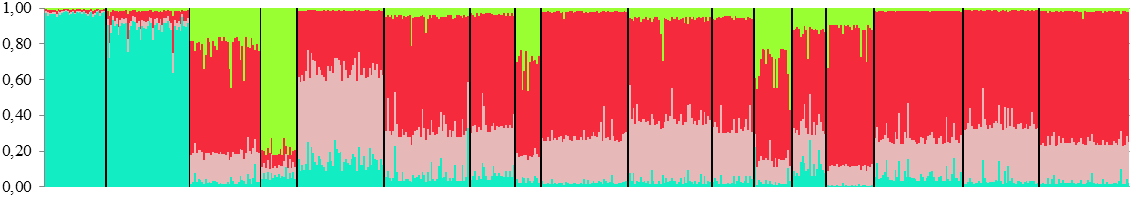

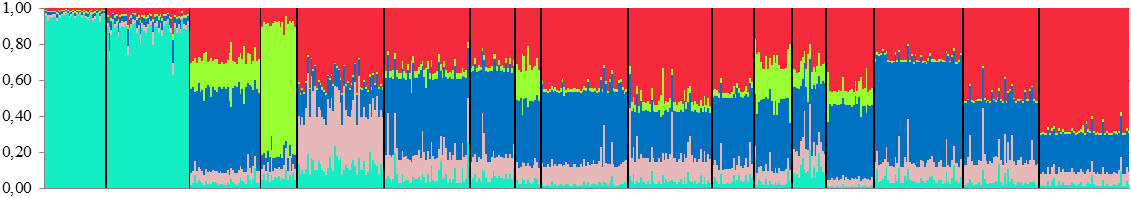


BAS-N BAS-S T NOR NS-E NS-C NS-S ICE IR-W IR-E IR-SW IR-SE ECH BB-FR BB-SE BB-SW SP-W
